# Sensory Evaluation, Physico-Chemical Properties, and Aromatic Profile of Pasteurised Orange Juice with Resistant Maltodextrin

**DOI:** 10.3390/foods12214025

**Published:** 2023-11-03

**Authors:** Elías Arilla, Javier Martínez-Monzó, Maria Simona Chiş, Anca Corina Fǎrcaş, Sonia Ancuţa Socaci, Pilar Codoñer-Franch, Purificación García-Segovia, Marta Igual

**Affiliations:** 1 Food Investigation and Innovation Group, Food Technology Department, Universitat Politècnica de València, Camino de Vera s/n, 46022 València, Spain; elarco@upv.es (E.A.); xmartine@tal.upv.es (J.M.-M.); pugarse@tal.upv.es (P.G.-S.); marigra@upvnet.upv.es (M.I.); 2 Deparment of Food Engineering, Faculty of Food Science and Technology, University of Agricultural Sciences and Veterinary Medicine of Cluj-Napoca, 3-5 Mănăştur Street, 400372 Cluj-Napoca, Romania; simona.chis@usamvcluj.ro; 3 Deparment of Food Science, Faculty of Food Science and Technology, University of Agricultural Sciences and Veterinary Medicine of Cluj-Napoca, 3-5 Mănăştur Street, 400372 Cluj-Napoca, Romania; anca.farcas@usamvcluj.ro (A.C.F.); sonia.socaci@usamvcluj.ro (S.A.S.); 4 Deparment of Pediatrics, Obstetrics and Gynecology, Universitat of València, Avenida de Blasco Ibáñez, No. 15, 46010 València, Spain; 5 Deparment of Pediatrics, Foundation for the Promotion of Health and Biomedical Research in the Valencian Region (FISABIO), University Hospital Dr. Peset, Avenida Gaspar Aguilar, No. 90, 46017 València, Spain

**Keywords:** prebiotics, fruit juices, sensory attributes, physico-chemical characteristics, aroma volatile compounds

## Abstract

The beneficial health effects of prebiotics have been demonstrated in numerous research papers. However, their incorporation into daily food remains unfamiliar to consumers. This work evaluates the effects of the addition of resistant maltodextrin (RMD) on the sensory attributes of pasteurised orange juice, together with the physico-chemical properties and the aromatic profile. RMD addition increased the sweetness and decreased the acidity and bitterness, resulting in a higher overall panellists’ rating of orange juice. It also proportionally increased °Brix together with density and decreased acidity. Colour changes were registered with higher RMD concentrations. Orange pulp presence affected the volume particle size distribution analysis, while RMD addition did not have any effect. The aroma volatile compounds were also analysed. Pulp-added samples showed a higher quantity of alcohol and aldehydes, whereas pulp-free samples registered higher terpene and terpenoid values. Ketones and acids were also quantified. RMD had a moderate impact on volatile compound quantifications, with the orange pulp presence playing a much more decisive role. A correspondence analysis was also performed to relate instrumental and sensory determinations for all samples. This work proves that the addition of RMD to orange juice is technologically feasible while also achieving a good response at the sensory level.

## 1. Introduction

Consumers’ consciousness towards a healthy diet in recent years has increased the demand for high-value food products, such as functional ingredients [1], aiming to improve the nutritional properties of conventional food and beverages. Within the functional ingredients, prebiotics emerge as a route of great interest to meet consumers’ needs due to their contribution to human health. Prebiotics are a group of dietary fibres whose selective fermentation results in specific changes in the composition and/or activity of the gastrointestinal microbiota, thus conferring benefits upon host health [2]. Substances such as inulin, fructo-oligosaccharides, gluco-oligosaccharides, lactulose, isomalto-oligosaccharides, or lactosucrose have been widely studied because of their prebiotic activity and their effects on human health [3,4]. In addition, resistant maltodextrin (RMD), which is a corn-based non-digestible fibre [5], is currently gaining popularity in clinical settings [6,7,8,9].

The functional foodstuff trend represents an innovation focus for the industry to develop new food products that go beyond basic nutrition and good taste [10,11,12]. As such, it entails technological challenges depending on the food matrix, processing, packaging, shelf life, and other aspects. For this reason, it is convenient to investigate the application of these substances to easily manageable matrices, such as beverages. Accordingly, orange juice is a suitable vehicle for this kind of functional ingredient because it already contains functional compounds [13] and because it is the most preferred fruit juice flavour among consumers [10,14]. In addition, prebiotics added to liquid matrices could be more effective in terms of health impact because they are usually easier to digest than solid foods. The application of a pasteurisation process to commercial orange juice is widespread. However, it is well known that thermal treatments cause a complex series of chemical reactions which directly affect the aroma volatile compounds, either losing the original aroma or developing foreign odours to fresh orange juice [15]. Since RMD is a starch-based ingredient, it could be used to retain and protect volatile compounds, therefore positively counteracting their loss through the evaporation process [16].

Packaging technologies and materials also have a role in the flavour retention of orange juice, reducing the intensity of aroma compounds [17]. For instance, van Willige et al. [18] highlighted that limonene, myrcene, and decanal amounts could be reduced during orange juice storage through the absorption of the PET packaging. Therefore, protecting or improving the original sensory attributes of heat-treated orange juice is an interesting challenge that needs to be further exploited.

To achieve social acceptance of prebiotic-added foods, it is of great relevance to elucidate the impact of such compounds on food products. The potential health benefits of prebiotics have been described in clinical trials [19]. However, the incorporation of functional ingredients into day-to-day foods is not yet commonplace [20]. Therefore, this work aimed to evaluate the sensory attributes of pasteurised orange juice with RMD, in addition to the physico-chemical properties and the aromatic profile. Analysing how functional ingredients influence the sensory properties of conventional foods is essential to improving consumer predisposition towards healthier eating habits.

## 2. Materials and Methods

### 2.1. Raw Materials

This study was conducted with freshly industrially squeezed orange juice supplied by Refresco Iberia S.A.U. (Valencia, Spain). All oranges used were of Spanish origin from different cultivars, such as Navelina, Salustiana, Navel, Navel Late, Lane Late, Navel Powell, and Valencia. Oranges were grown in conventional planting conditions. The orange juice extraction followed the standard manufacturing procedure: reception of oranges from suppliers, surface cleaning of the orange peel, classification according to fruit diameter, juice extraction in industrial juicers (Citrus Juice Extractor Model 593, JBT, Chicago, IL, USA), and sieving to separate the residual pulp from orange juice. Orange juices from different cultivars were mixed to ensure a homogeneous quality, as is commonly performed in the juice industry. RMD (Fibersol-2) added to the juice was purchased from ADM/Matsutani, LLC (Decatur, IL, USA). Frozen pasteurised orange pulp was provided by a local fruit processing company (Zumos Valencianos del Mediterráneo, Valencia, Spain).

### 2.2. Sample Preparation and Pasteurisation

Eight samples of orange juice were prepared. Four were orange juice with pulp (OJP), and the other four were orange juice without pulp (OJWP). Fresh orange juice was directly collected from the industrially squeezed lines. Orange pulp (2.5%) was added to the OJP samples. Pulp content was homogenised using a stirrer (LH Overhead Stirrer, VELP Scientifica, Italy) by applying 200 rpm for 5 min. Increasing RMD concentrations (2.5, 5, and 7.5%) were mixed into OJP and OJWP samples. Thus, for a final beverage portion of 200 mL, 5, 10, or 15 g of RMD, respectively, would be ingested, enough to display functional effects according to other studies [7,8,9,10]. Control samples without RMD addition (OJP0 and OJWP0) were also prepared, and they complied with the European Fruit Juice Association orange juice guidelines [21], so no adulteration or deviation occurred during the juice extraction. To properly dissolve RMD in the fresh orange juice, the same stirrer was used at 200 rpm for 15 min. Finally, all samples were pasteurised (Fruchtsaftdispenser, Mabo Steuerungselemente GmbH, Germany) at 85 °C for 10 s, and were hot-filled into 250 mL polyethylene terephthalate (PET) bottles. After the heat treatment, all bottles were immersed in a cold water bath (<10 °C) for 30 min to cool down.

### 2.3. Sensory Analysis

An expert panel of 15 members, 6 men and 9 women, performed a sensory analysis of the orange juice samples. All members of the panel, aged between 28 and 55 years old, volunteered from the Quality Control department of Refresco Iberia S.A.U., an orange juice manufacturer and bottling company. Therefore, they had professional experience testing orange juice. They evaluated the colour, aroma, sweetness, acidity, bitterness, mouthfeel (softness, thickness), off-flavour, and overall rating as the attributes of the orange juice samples using a 9-point hedonic scale (9 = like extremely; 1 = dislike extremely) [22].

During the test session, panellists worked isolated in individual booths. All samples were presented in a random order to the panellists at 20 °C under normal lighting conditions in 50 mL cups, and a 3-digit random number was placed on them, identifying each sample. Water at room temperature was given to the panellists to clean the palate before trying the next sample. A testing sheet for each sample identified with the 3-digit random number was given to each panellist to record the results of the sensory evaluation. During the session, the panellists evaluated all 8 samples (with and without orange pulp addition and with and without RMD addition).

All participants gave their informed consent before engagement in this study. For data confidentiality purposes, each member of the panel was assigned a random three-digit code. Data were treated anonymously and following the European General Data Protection Regulation (Regulation E.C. (2016). No 679/2016 of the European Parliament and of the Council of 27 April 2016).

### 2.4. Physico-Chemical Determinations

#### 2.4.1. °Brix, pH, Acidity, and Density

Total soluble solids (°Brix) were measured with refractometry (Abbemat 200, Anton Paar, Austria). pH determination was conducted using a Basic 20 pH meter (Crison, Barcelona, Spain). Acidity, expressed as grams of citric acid per 100 mL (gCA/100 mL), was determined using a DL53 acid titrator (Mettler Toledo, Greifensee, Switzerland). Density was obtained using a densimeter (DMA 5000, Anton Paar, Austria). All determinations were performed in triplicate, following AOAC guidelines [23].

#### 2.4.2. Particle Size

Particle size distribution of juices was determined with the laser diffraction method and Mie theory [24], using a particle size analyser (Malvern Instruments Ltd., Mastersizer 2000, UK) equipped with a wet sample dispersion unit (Malvern Instruments Ltd., Hydro 2000 MU, UK). Laser diffraction reports the volume of material of a given size, since the light energy reported by the detector system is proportional to the volume of material present. The Mie theory requires information on the sample and the dispersant optical properties. For orange juice, the particle refraction and absorption were 1.52 and 0.1, respectively, and the water refraction index was 1.33. The sample was dispersed in distilled water and pumped through the optical cell under moderate stirring (1800 rpm) at 20 °C. The volume (%) against particle size (in µm) was obtained, and the size distribution was characterised by the volume mean diameter (D[4,3]). The standard percentile d(0.1), or particle size below which 10% of the sample lies, and d(0.9), or particle size below which 90% of the sample lies, were also considered for juice characterisation.

#### 2.4.3. Colour Measurement

The sample colour was measured using a colourimeter (Konica Minolta CM-700 d/600 d series, Tokyo, Japan) with a standard illuminant D65 and a visual angle of 10°. Results were obtained in terms of L* (brightness: L* = 0 (black), L* = 100 (white)), a* (−a* = greenness, +a* = redness), and b* (−b* = blueness, +b* = yellowness), according to the CIELab system [25]. Total colour differences (ΔE) were calculated by comparing each sample with RMD with its corresponding control (OJWP0 or OJP0).

### 2.5. Analysis of Aroma Volatile Compounds with ITEX/GS-MS

The extraction and analysis of volatile compounds were performed according to Igual et al. [26] using the in-tube extraction technique (ITEX) followed by their separation and identification with gas chromatography–mass spectrometry (GC-MS), using a GC-MS QP-2010 model (Shimadzu Scientific Instruments, Kyoto, Japan) equipped with a Combi-PAL AOC-5000 autosampler (CTC Analytics, Zwingen, Switzerland) and a capillary column (ZB-5 ms, 30 m × 0.25 mm i.d. × 0.25 µm, Phenomenex, Torrance, CA, USA). For the volatile extraction step, a hermetically sealed headspace vial containing 0.5 g of sample was incubated at 60 °C under continuous agitation for 10 min. After the incubation, from the headspace phase, the volatile compounds were adsorbed (aided by the headspace syringe) repeatedly (15 strokes) into a porous polymer fibre microtrap (ITEX-2TRAPTXTA, Tenax TA 80/100 mesh, ea). The extraction of the volatile compounds and their thermal desorption and injection into the GC-MS injector were performed automatically using the Combi-PAL AOC-5000 autosampler. The following parameters were used for the column oven: from 38 °C, the temperature rose to 110 °C and then to 250 °C at 4 °C/min and 20 °C/min, respectively, and the final temperature was held for 5 min. The identification of the samples’ volatiles was based on their mass spectra using the software’s NIST27 and NIST147 mass spectra libraries and was verified and compared with retention indices drawn from databases [27,28]. Results are expressed as a relative percentage of the total peak area.

### 2.6. Statistical Analysis

Analysis of variance (ANOVA) was applied with a confidence level of 95% (*p* < 0.05) to evaluate the differences among samples. Furthermore, a correspondence analysis (CA) among sensory attributes of juices was conducted with a 95% of significance level. A multiple factor analysis (MFA) was also carried out with the mean values of all data (instrumental and sensorial) to explore the relationship between them. Each sample is represented by 8 points, grouping sensory and instrumental variables studied in the samples. The consensus representation that considers the 8 variables simultaneously is also represented for each juice. From the sensory analysis, sweetness, acidity, and bitterness were grouped into sensory taste variables. On the other hand, off-flavours and flavour appeal were grouped into a variable named sensory flavour. Colour and mouthfeel in the sensory test have been kept individually as sensory colour and sensory texture, respectively. The variables obtained from the instrumental measurements have been grouped as follows: L*, a*, and b* as the instrumental colour; °Brix and titratable acidity as the instrumental flavour; density and particle size parameter D[4,3] as the instrumental texture; and the different groups of analytically determined aromatic compounds as the instrumental aroma. Statgraphics Centurion XVII software, version 17.2.04 (Statgraphics Technologies, Inc., The Plains, VA, USA), and XLSTAT statistical software version 2021 were used [29].

## 3. Results and Discussion

### 3.1. Sensory Evaluation

**Figure 1:**
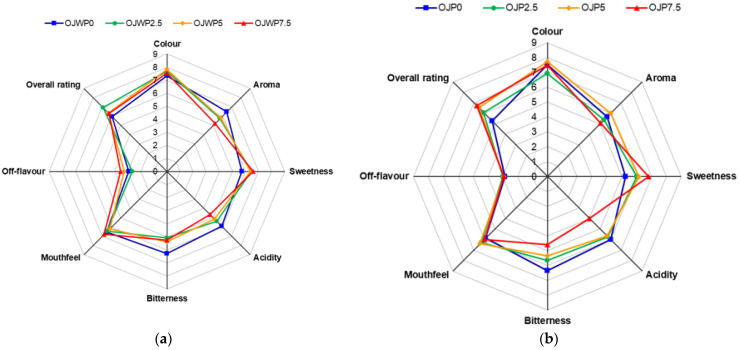
Mean values scores of the different sensory attributes evaluated in pasteurised orange juice with or without pulp and 0–7.5 % of RMD addition. (**a**) OJWP, orange juice without pulp; (**b**) OJP, orange juice with pulp. Concentric octagonal isolines show the axis tick marks.

Figure 1 shows the mean value scores of each sample without orange pulp (a) and with orange pulp (b) for each evaluated sensory attribute. In general terms, the most noticeable changes occurred in the sensory parameters related to smell and taste, such as aroma, sweetness, acidity, and bitterness, which affected the overall rating of the samples in both OJWP (a) and OJP (b) samples, respectively. OJWP0 was marked as having the most appealing aroma of all samples, with a mean value of 6.47 (2.17). OJWP0 also scored the highest value for acidity, with a mean value of 5.93 (1.44), and bitterness, with a mean value of 6.27 (1.62), and was ranked the lowest in terms of sweetness, with a mean value of 5.73 (1.91). OJP0 had similar scores to OJWP0, which led to the lowest overall rating scores for both standard samples, with mean values of 5.27 (1.39) and 5.93 (1.53), respectively. This was expected since the standard samples consist only of pasteurised orange juice (OJWP0) and pasteurised orange juice with pulp (OJP0), without any addition of RMD.

RMD addition to OJWP and OJP samples improved almost all the sensory attributes, except for the aroma, which obtained slightly lower scores. For example, samples with a 7.5% RMD concentration were scored mean values of 5.07 (2.19) for the pulp-added orange juice and 5.20 (1.90) for the pulp-free orange juice. This could be due to RMD addition, which led to a reduction in the orange juice content in the final sample, and therefore, an aromatic loss could be expected. Moreover, RMD-added OJP samples did not show any additional off-flavour compared to the standard OJP0 sample. In contrast, RMD-added OJWP samples presented more off-flavours, especially at high RMD concentrations, where the 7.5% sample had a mean value of 3.53 (2.10), in comparison to the OJWP0 with a mean value of 2.93 (1.79). Rega et al. [30] stated that orange pulp strongly influences flavour release in orange juice and increases the fresh orange juice character. Thus, orange pulp seems to play a role in masking possible off-flavours derived from RMD addition to orange juice. In addition, the sweet/acid ratio has been identified as a basic precept when judging the sensory quality of many fruit-based products [31]. RMD incorporation increased the sweetness scores and decreased the acidity and bitterness scores, with this effect being higher at higher RMD concentrations and clearer in the OJP samples. Thus, OPJ7.5 had mean values of 6.80 (1.42), 4.00 (1.60), and 4.60 (1.24) for sweetness, acidity, and bitterness, respectively. This resulted in a higher overall rating of the RMD-added samples. This can be clearly observed in both OJP7.5 and OJWP7.5, which scored mean values of 6.73 (1.87) and 6.27 (1.58), respectively. This contrasts with the study by Luckow and Delahunty [32], who found that consumers prefer the sensory characteristics of conventional orange juices to their functional (probiotic and prebiotic) counterparts. This could be an indicator that RMD is more suitable for developing sensory-attracting functional foods in comparison with other functional ingredients.

The Tukey’s HSD (Honestly Significant Difference) method applied to the sum of ranks was used to perform a multiple comparison among the samples. The calculated Tukey’s HSD value, according to assay conditions, was 40.7. When the difference between the sums of rank of each pair of samples, for each attribute, was greater than 40.7, significant differences between paired samples were assumed. Significant differences were not observed among the eight samples studied (*p* > 0.05). Therefore, the expert judges could not appreciate the differences among the samples for any attribute that showed a significant effect.

**Figure 2:**
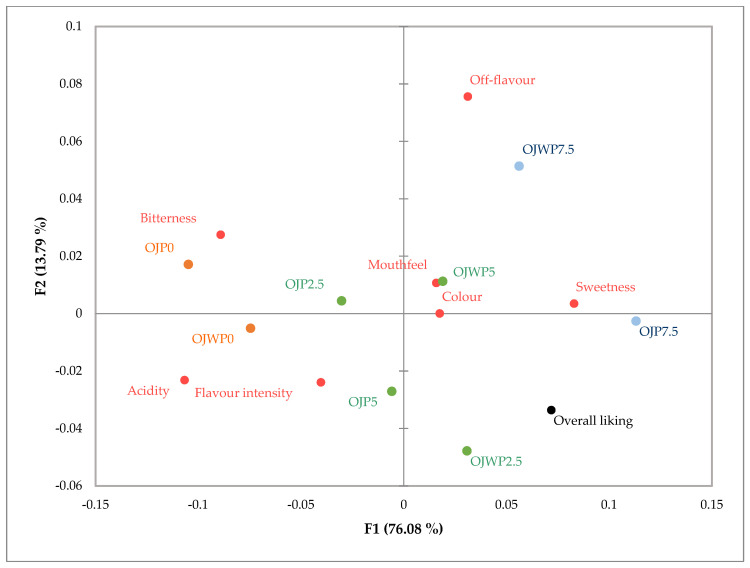
Correspondence analysis. Representation of sensory attributes and samples tested in the normalised plane defined by the two factors, explaining the variability in the results of the sensory analysis. OJWP, orange juice without pulp; OJP, orange juice with pulp; 0–7.5 % of RMD addition. Colours orange, green, and blue indicate different groups of clusters in CA.

A correspondence analysis was carried out to relate the samples by means of the different juices with all the attributes evaluated and the assessors’ preferences. From this analysis, two factors were obtained that explained 90% of the variability in the results (Figure 2). The first factor (F1) explained 76.08% of the variability, and the second (F2) explained 13.79%. Figure 2 shows the projection in the plane of the juices and attributes derived from the correspondence analysis. Samples are mainly ordered from left to right in the graph, from the lowest to the highest RMD concentration on F1. OJP0 was identified as more bitter, whilst OJWO was related to acidity and flavour intensity. The OJWP5 sample was favourably evaluated for its colour and mouthfeel, and juices with higher concentrations of RMD were identified as the sweetest. Furthermore, sweetness is the sensory attribute that has the most weight on overall liking.

### 3.2. Physico-Chemical Properties

The °Brix content of orange juice comes from its sugar content, mainly fructose, sucrose, and glucose. Also, acidity is a result of its citric acid content. The °Brix/acid ratio plays a major role in the sensory properties of orange juice. For this reason, it is commonly used as a juice quality and fruit maturity indicator [33]. They are also measured to ensure non-deviation during the juice extraction process, in accordance with the AIJN [30]. OJWP0 had almost the same (*p* > 0.05) °Brix as OJP0 (Table 1). RMD addition proportionally increased (*p* < 0.05) the °Brix values of orange juice in both OJWP and OJP samples. This phenomenon was expected as RMD displays good dissolving properties in water [5]. Contrariwise, orange pulp, an insoluble fibre, did not have a significant (*p* > 0.05) impact on °Brix. Unlike with °Brix, adding higher concentrations of RMD to orange juice proportionally decreased (*p* < 0.05) its acidity. This could be explained since RMD addition reduced the quantity of raw orange juice and therefore citric acid. Similar results were obtained in previous work on RMD addition to orange juice [34,35,36]. Other studies on prebiotic-added fruit-based beverages also showed the same behaviour [37,38,39]. Thus, adding RMD to orange juice helped to achieve a favourable sweet/acid balance, as RMD-added samples obtained higher overall rating scores (Figure 1). RMD incorporation into orange juice did not affect (*p* > 0.05) the pH, which ranged between 3.67 and 3.71 in all samples. The potential technological applications of prebiotics not only for a nutritious upgrade but also for sensory and physico-chemical improvements in conventional food have been discussed [40].

The colour of orange juice, which is mainly due to carotenoid pigments, plays an important role as a quality indicator and is key to consumer acceptance [41]. RMD addition at higher concentrations significantly decreased L* values (*p* < 0.05) (Table 1). Therefore, all orange juices turned slightly darker when the RMD concentration was raised. This is probably because adding RMD reduced the orange juice content in the finished samples. Orange pulp showed a small but significant (*p* < 0.05) effect on the L* values, as all OJP RMD-added samples showed higher L* values than the free-pulp samples at the same RMD concentrations. In addition, OJWP samples obtained, in general, lower a* values than OJP samples, meaning that OJP samples were slightly redder than OJWP samples. This could be an indicator that pulp incorporation into orange juice could add carotenoids to orange juice. Accordingly, in a past study on orange juice bioactive compounds, pulp-added orange juice presented higher carotenoid content than pulp-free orange juice [35]. The b* value was barely affected by orange pulp presence. Additionally, RMD incorporation into orange juice decreased (*p* < 0.05) the L* and b* values, meaning that samples lost brightness and yellowness, especially at higher RMD concentrations. The a* values were also affected by RMD but not in a significant way. Higher RMD concentrations caused (*p* < 0.05) colour changes in both OJWP and OJP samples, mainly in pulp-free samples. According to Bodart et al. [42], if the ΔE is larger than 3 units, colour changes may start to be perceptible by the human eye. Instrumentally, it was proven that the addition of pulp helped to better retain the original colour of the orange juice in the RMD-added samples, although panellists did not find evident colour differences among all samples during sensory evaluation (Figure 1).

**Figure 3:**
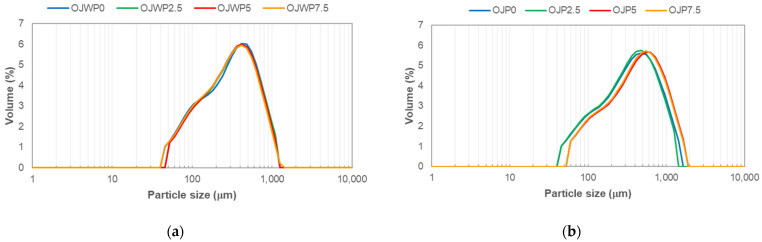
Volume particle size distributions (representative curves) of pasteurised orange juice with or without pulp and 0–7.5 % of resistant maltodextrin. (**a**) OJWP, orange juice without pulp; (**b**) OJP, orange juice with pulp.

The analysis of the volume particle size distribution for all OJWP (Figure 3a) and OJP (Figure 3b) samples indicated that both followed a similar trend. However, pulp-free samples had less data dispersion. Pulp-added samples, on the contrary, seemed to show more data dispersion. This is likely due to the addition of orange pulp rather than the addition of RMD, since pulp is an insoluble solid of variable size, while RMD has good water-soluble properties and, therefore, should barely affect the volume particle size distribution. Table 2 summarises the mean values (and standard deviations) of volume mean diameter D[4,3] and the standard percentiles d(0.1), d(0.5), and d(0.9). The particle size of OJP samples presented significantly greater volume mean diameter (*p* < 0.05) than OJWP samples exclusively because of orange pulp addition. Also, RMD addition to orange juice hardly had an impact (*p* > 0.05) on the volume mean diameter mainly because of its water-soluble properties.

Juice density is also an important quality control parameter in the juice industry [32]. The OJWP0 and OJP0 samples presented the same (*p* > 0.05) density values. However, adding higher concentrations of RMD to orange juice proportionally increased the density values (*p* < 0.05). In addition, the RMD-added OJP samples presented slightly higher (*p* < 0.05) density values compared to the OJWP samples at the same RMD concentrations. The effect of RMD on orange juice density was expected as it completely dissolved in water. Moreover, density values and °Brix (soluble solids) have traditionally presented a clear relationship in matrices that practically only contain soluble solids, such as fruit juices. As a result, regression models have been proposed [43,44]. On the contrary, insoluble solids such as cloud and pulp contribute little to the density measurement [32]. Accordingly, the °Brix (Table 1) and density values (Table 2) of both OJP and OJWP RMD-added samples proportionally followed the same trend in this study.

### 3.3. Aroma Volatile Compounds

The taste of food is mainly based on the flavours and aromas of ingredients [45]. Flavour has a direct influence on consumer satisfaction, directly influencing consumption. In the present study, the aroma volatile compounds were divided into five groups: alcohols, aldehydes, terpenes and terpenoids, ketones, and acids. Table 3 shows the analysis of volatile compounds of these groups for all OJWP and OJP samples. The OJP samples reported higher (*p* < 0.05) alcohol content than the OJWP samples, mainly because the content of 1-terpinen-4-ol increased (*p* < 0.05) in pulp-added samples. 1-terpinen-4-ol has been considered by some authors as an index of degradation of the aroma in the orange juice or as an indicator of the age of orange juice [46]. OJP0 showed the highest (*p* < 0.05) amount of 1-terpinen-4-ol. RMD addition slightly decreased (*p* < 0.05) 1-terpinen-4-ol content in all OJWP and OJP samples, with this effect being clearer in OJWP samples. Keeping 1-terpinen-4-ol at the lowest possible levels is desirable, as it has been reported as one of the predominant compounds contributing to storage off-flavour development in citrus juices [47]. Moreover, 1-terpinen-4-ol comes from the acid-catalysed hydration of limonene and linanool [48], which could be enhanced when the °Brix/acid ratio is low, in acidic conditions. Therefore, a decrease in 1-terpinen-4-ol could be expected since adding RMD to orange juice increased °Brix and decreased citric acid content (Table 1).

Aldehydes are secondary metabolites formed during the ripening and maturation period of orange fruit [49]. The main representant within the aldehydes was octanal, followed by decanal and nonanal. Perez-Cacho and Rouseff [50] reported that these compounds were present at 45 (octanal), 22 (nonanal), and 10 (decanal) times greater concentrations in mechanically squeezed orange juice compared to hand-squeezed because industrial juice extraction practices introduce relatively high levels of peel oil. Brat et al. [51] also quantified greater amounts of octanal, nonanal, and decanal in orange pulp than in orange juice. This explains why pulp-added samples showed higher (*p* < 0.05) amounts of octanal, nonanal, and decanal, and subsequently higher (*p* < 0.05) total values of aldehydes, than OJPW samples. Furthermore, RMD addition to OJWP samples decreased (*p* < 0.05) total aldehyde content. Interestingly, it displayed the opposite effect in pulp-added orange juice, with OJP5 showing the highest (*p* < 0.05) value of total aldehydes among all samples. Therefore, RMD addition to pulp-added orange juice seems to retain higher aldehyde concentrations, especially of octanal, which along with nonanal and decanal has been related to a more intense orange-like/green note in the overall aroma profile of juice from Valencia variety oranges [52].

The terpenes and terpenoids group reached the largest amount of all five volatile compound groups. Limonene and β-myrcene were the main compounds in this group, with higher (*p* < 0.05) quantification in the OJWP samples than in the OJP samples in both cases. The presence of limonene and β-myrcene at these values seems to be related to orange juice extraction, as industrial processes apply higher manufacturing pressures compared to manual hand-squeezing processes, therefore leading to higher orange peel oil extraction [14,53]. Pulp incorporation implied a reduction in orange juice in OJP samples. As a result, pulp-free samples obtained higher (*p* < 0.05) total values of terpenes and terpenoids, ranging between 84.38% and 86.74%, whilst pulp-added samples registered values in the range of 81.07% to 82.19%. Although limonene concentration could be influenced by several factors, such as orange maturity stage, origin, variety, way of harvesting, and juice processing conditions [54], it has been also found to be one of the predominant volatile compounds in other orange juice studies [48]. Despite its high concentration, the contribution of limonene to orange aroma is considered the most conflicted of any of the volatiles in orange juice. In fact, Perez-Cacho and Rouseff [14] stated that limonene does not display a key impact in orange juice, although it is a necessary component of any orange juice odour model. In other study, they also hypothesised that limonene could work as a “lifting agent” for other volatiles, in a similar way as ethanol does in wine [50].

In addition to limonene and β-myrcene, α-pinene was the next highest quantified volatile compound in the terpenes and terpenoids group, especially in the pulp-added samples. Brat et al. [51] reported a higher amount of α-pinene in orange pulp than in orange juice. Therefore, this seems to be exclusively due to orange pulp incorporation. RMD addition slightly increased (*p* > 0.05) the total values of terpenes and terpenoids in pulp-free samples. However, higher RMD concentration did not cause a higher impact. Moreover, it did not have an effect (*p* > 0.05) on pulp-added samples.

From the ketones group, no difference (*p* > 0.05) was observed between OJP0 and OJWP0. RMD hardly increased (*p* > 0.05) total ketone quantification in pulp-free samples. However, in pulp-added samples, RMD incorporation did had a significant effect by increasing (*p* < 0.05) total ketone values. Ketones have been related to the aromatic quality of orange juice [45]. Moreover, from the acids group, the biggest amount was identified in OJWP0 and OJP0 in an increasing manner (*p* < 0.05), with butanoic acid-ethyl ester and acetic acid-octyl ester being the mainly identified acids. This could be correlated with the fact that, during sensory analysis, panellists identified control samples with higher acidity. Adding higher RMD concentrations reduced (*p* < 0.05) total acid values, probably because the addition of RMD implied a reduction in orange juice in the final samples.

Consumers expect high-quality juices, with sensory properties like those found in unprocessed fresh juices. However, pasteurisation is known to affect aroma release in a negative way [14]. This seems to be related to interactions such as the polymerisation of proteins and pectin during heat treatment [55]. Orange juice can be considered as a multiphase system of an aqueous phase and a water-insoluble phase comprising both cloud and pulp, which contains large amounts of cell wall polysaccharides and can be a source of pectin [52]. Therefore, pulp particles could enhance such chemical reactions during the orange juice pasteurisation process.

### 3.4. Instrumental and Sensory Correlations

**Figure 4:**
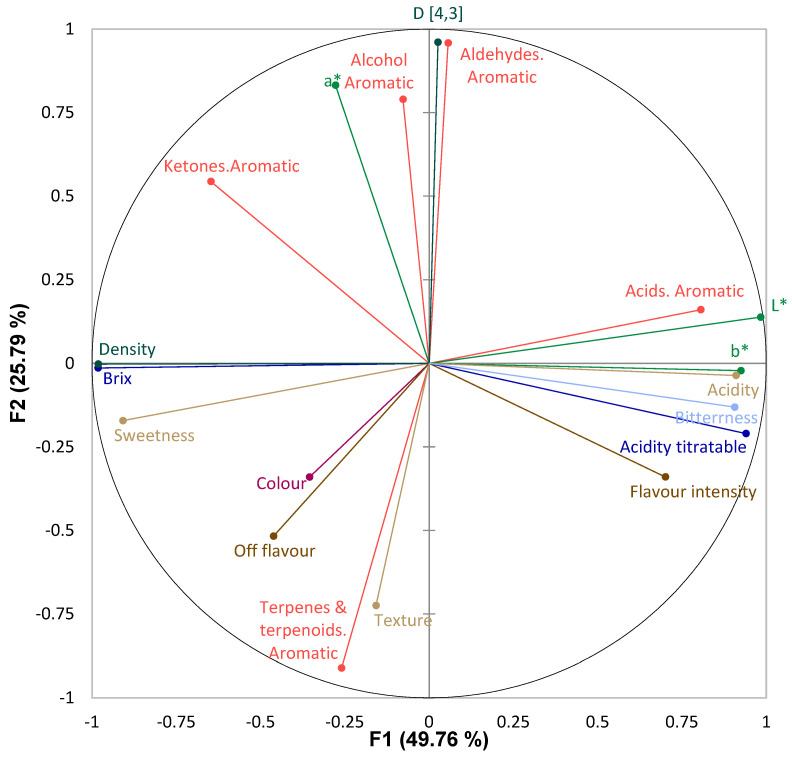
Correlation circle of the response variables.

MFA was used to study the influences of all the different types of variables and to relate the results from instrumental and sensory determinations for the evaluated juices. The MFA constructs a sensory map in two dimensions. Factors 1 (F1) and 2 (F2) explained 75.55% of the data variability (Figure 4). The sensory acidity detected by panellists corresponds to the instrumentally measured acidity. In turn, the sensory sweetness reported by the panellists is also close to the measured °Brix. The parameter D[4,3] of the particle size determination and the perceived mouthfeel of the juices are related in F2 but in the opposite way. The sensorily evaluated off-flavours are close to the instrumentally quantified terpenes and terpenoids, and the flavour intensity is related to the acids of the determined aroma compounds.

Figure S1 shows the superimposed MFA representation of the juices. Each sample is represented by eight points grouping sensory and instrumental variables studied in the samples. The consensus representation that considers the eight variables simultaneously is also represented for each juice. From the sensory analysis, sweetness, acidity, and bitterness have been grouped into sensory taste variables. On the other hand, off-flavours and flavour intensity have been grouped into a variable named sensory flavour. Colour and mouthfeel in the sensory test have been kept individually as sensory colour and sensory texture, respectively. The variables obtained from the instrumental measurements have been grouped as follows: L*, a*, and b* as the instrumental colour; °Brix and titratable acidity as the instrumental flavour; density and particle size parameter D[4,3] as the instrumental texture, and the different groups of analytically determined aromatic compounds as the instrumental aroma.

As shown in Figure 4 and Figure S1, there was a significant difference between juices with and without pulp, according to F2. The juices with pulp are closer to larger particle sizes and aromatic compounds determined in this study. On the other hand, juices without pulp are closer to the sensory attribute of texture in the mouth and to off-flavours. In addition, samples with the highest RMD concentrations are close to the sensory sweetness and °Brix determined, as well as to the density. However, standard samples without RMD addition are close to the sensory evaluation of flavour, acidity, and bitterness and to instrumentally determined L*, a*, titratable acidity, and acid aroma compounds.

The superimposed representation of the samples in the MFA made it possible to evaluate the proximity between the studied variables for all samples. Observing the underlying structure of the instrumental and sensory variables and their proximities, it can be inferred that variables such as sensory colour and flavour or instrumental aroma influence the position of the samples in the plane. The most significant differences in the samples are generally produced by the variables mentioned above, and the rest of the variables studied in the MFA present different distances for each sample. Therefore, these differences affect each sample differently. This trend is also observed in the RV coefficients obtained from the MFA (Table 4). The RV coefficient is a multivariate statistic ranging from 0 (full disagreement) to 1 (perfect agreement) [56]. The RV coefficients range from 0.6 to 0.9. Sensory colour, flavour, and instrumental aroma showed the lowest RV values against the other variables, while the rest of studied variables showed higher RV coefficients, above all instrumental colour. This indicates that sensory colour and flavour or instrumental aroma are important variables in the evaluation of the studied juices.

## 4. Conclusions

Despite the growing background on the beneficial effects of prebiotics on health, their incorporation into day-to-day foods remains unfamiliar to consumers. As an approach, this work provides evidence that the addition of RMD to pasteurised orange juice is technologically feasible while also achieving a good response at the sensory level, even at high RMD concentrations, according to expert panellists. RMD improved almost all sensory attributes, leading to higher overall rating scores than RMD-free control samples. Also, RMD addition to orange juice displayed a very similar impact on the physico-chemical properties of orange juice as was previously found by our group. RMD addition had a moderate effect on volatile compound quantification, whereas the presence of orange pulp played a much more decisive role by increasing 1-terpinen-4-ol, octanal, nonanal, decanal, and α-pinene and decreasing limonene and β-myrcene.

Therefore, adding RMD may be of interest to upgrade the organoleptic acceptability of conventional fruit juices, such as orange juice. This can favour the consolidation of prebiotic addition to day-to-day foods in consumers’ diet. It would be of interest to perform a sensory analysis of RMD-added orange juice with consumers to tackle this challenge.

## Figures and Tables

**Table 1:** Mean values (and standard deviations) of °Brix, pH, acidity (g_CA_/100 mL), and colour coordinates (L*, a*, and b*) of pasteurised orange juice and total colour differences (ΔE).

Sample	°Brix	pH	Acidity	L*	a*	b*	ΔE
OJWP0	12.3 (0.2) ^d^	3.67 (0.02) ^b^	0.920 (0.002) ^a^	40.68 (0.13) ^a^	−1.54 (0.13) ^d^	29.6 (0.3) ^a^	-
OJWP2.5	14.4 (0.2) ^c^	3.67 (0.02) ^b^	0.882 (0.002) ^c^	37.89 (0.16) ^d^	−1.29 (0.07) ^bc^	28.70 (0.07) ^c^	2.95 (0.13) ^e^
OJWP5	16.5 (0.2) ^b^	3.67 (0.02) ^b^	0.861 (0.002) ^e^	36.80 (0.12) ^e^	−1.48 (0.09) ^d^	27.4 (0.4) ^d^	4.5 (0.2) ^c^
OJWP7.5	18.7 (0.2) ^a^	3.67 (0.02) ^b^	0.838 (0.002) ^g^	35.12 (0.02) ^g^	−1.51 (0.02) ^d^	26.43 (0.12) ^e^	6.41 (0.05) ^a^
OJP0	12.2 (0.2) ^d^	3.70 (0.02) ^a^	0.891 (0.002) ^b^	40.13 (0.10) ^b^	−1.35 (0.12) ^c^	29.4 (0.4) ^ab^	-
OJP2.5	14.4 (0.2) ^c^	3.68 (0.02) ^b^	0.872 (0.002) ^d^	38.84 (0.13) ^c^	−1.240 (0.002) ^ab^	28.76 (0.15) ^bc^	1.46 (0.07) ^f^
OJP5	16.5 (0.2) ^b^	3.71 (0.02) ^a^	0.849 (0.002) ^f^	37.9 (0.3) ^d^	−1.350 (0.014) ^c^	26.5 (1.2) ^e^	3.7 (0.5) ^d^
OJP7.5	18.8 (0.2) ^a^	3.68 (0.02) ^b^	0.832 (0.002) ^h^	35.69 (0.13) ^f^	−1.16 (0.05) ^a^	26.5 (0.5) ^e^	5.3 (0.2) ^b^

The same letter in superscript within the column indicates homogeneous groups established by ANOVA (*p <* 0.05). OJWP, orange juice without pulp; OJP, orange juice with pulp; 0–7.5 % of RMD addition; L*, brightness; a*, greenness–redness tones; b*, blueness–yellowness tones; ΔE, total colour differences.

**Table 2:** Mean values (and standard deviations) of volume mean diameter (μm) D[4,3], standard percentiles (μm) d(0.1), d(0.5), and d(0.9), and density (g/cm^3^) of pasteurised orange juice.

Sample	D[4,3]	d(0.1)	d(0.5)	d(0.9)	Density
OJWP0	302 (13) ^cd^	25.6 (1.6) ^c^	245 (10) ^cd^	665 (30) ^c^	1.0487 (0.0002) ^g^
OJWP2.5	307 (4) ^c^	26.1 (1.2) ^c^	247 (5) ^c^	677 (8) ^c^	1.0575 (0.0003) ^f^
OJWP5	299 (14) ^cd^	23 (2) ^e^	242 (11) ^cd^	657 (31) ^c^	1.0659 (0.0009) ^d^
OJWP7.5	286 (12) ^d^	19.9 (1.2) ^f^	230 (9) ^d^	632 (28) ^c^	1.0757 (0.0004) ^b^
OJP0	427 (26) ^ab^	43 (3) ^ab^	332 (18) ^ab^	963 (66) ^a^	1.0487 (0.0005) ^g^
OJP2.5	410 (37) ^b^	41 (4) ^b^	324 (29) ^b^	916 (87) ^b^	1.0579 (0.0002) ^e^
OJP5	433 (20) ^a^	41 (3) ^ab^	339 (18) ^a^	974 (47) ^a^	1.0670 (0.0003) ^c^
OJP7.5	427 (25) ^ab^	44 (4) ^a^	335 (20) ^ab^	957 (62) ^ab^	1.0762 (0.0007) ^a^

The same letter in superscript within the column indicates homogeneous groups established by ANOVA (*p* < 0.05). OJWP, orange juice without pulp; OJP, orange juice with pulp; 0–7.5 % of RMD addition; D[4,3], volume mean diameter; d(0.1), standard percentile which encompasses the particles whose size is below 10% of the sample; d(0.5), standard percentile which encompasses the particles whose size is below 50% of the sample; d(0.9), standard percentile which encompasses the particles whose size is below 90% of the sample.

**Table 3:** Mean values (and standard deviations) of aroma volatile compound quantification (relative % from total peaks area) of pasteurised orange juice.

**Alcohols**	**OJWP0**	**OJWP2.5**	**OJWP5**	**OJWP7.5**	**OPJ0**	**OPJ2.5**	**OPJ5**	**OPJ7.5**
1-Octanol	0.09(0.03) ^bc^	0.11(0.03) ^bc^	0.07(0.03) ^c^	0.07(0.01) ^c^	0.21(0.02) ^a^	0.22(0.02) ^a^	0.18(0.01) ^a^	0.11(0.02) ^b^
3-methyl-1-Butanol,	0.07(0.02) ^b^	0.06(0.02) ^bc^	0.06(0.03) ^bc^	0.07(0.02) ^b^	0.16(0.02) ^a^	0.02(0.01) ^d^	n.d.	0.03(0.02) ^cd^
2-methyl-1-Butanol	n.d.	0.04(0.02) ^b^	n.d.	n.d.	n.d.	n.d.	0.35(0.02) ^a^	n.d.
1-Terpinen-4-ol	0.69(0.02) ^c^	0.60(0.02) ^d^	0.57(0.02) ^d^	0.49(0.02) ^e^	1.03(0.02) ^a^	0.88(0.02) ^b^	0.87(0.02) ^b^	0.94(0.02) ^b^
cis-p-Mentha-2,8-dien-1-ol		0.13(0.02) ^c^	0.16(0.03) ^bc^	0.15(0.02) ^bc^	0.17(0.02) ^b^	0.14(0.02) ^bc^	0.28(0.02) ^a^	0.25(0.02) ^a^
2-Cyclohexen-1-ol, 2-methyl-5-(1-methylethenyl)-, cis-//cis-Carveol	0.34(0.03) ^bc^	0.24(0.02) ^d^	0.29(0.04) ^c^	0.31(0.02) ^c^	0.23(0.02) ^d^	0.12(0.02) ^e^	0.39(0.02) ^a^	0.37(0.02) ^ab^
2-Cyclohexen-1-ol, 2-methyl-5-(1-methylethenyl)-, trans-//trans-Carveol	n.d.	0.08(0.02) ^bc^	0.06(0.01) ^cd^	0.07(0.02) ^bc^	0.03(0.02) ^d^	0.03(0.02) ^d^	0.11(0.01) ^ab^	0.12(0.02) ^a^
**Total**	1.19(0.10) ^d^	1.26(0.15) ^d^	1.25(0.16) ^d^	1.16(0.11) ^d^	1.83(0.12) ^b^	1.41(0.11) ^c^	2.18(0.10) ^a^	1.82(0.12) ^b^
**Aldehydes**	**OJWP0**	**OJWP2.5**	**OJWP5**	**OJWP7.5**	**OPJ0**	**OPJ2.5**	**OPJ5**	**OPJ7.5**
Dodecanal	0.07(0.02) ^bc^	0.05(0.02) ^c^	0.06(0.02) ^c^	0.05(0.02) ^c^	0.12(0.02) ^a^	0.10(0.02) ^ab^	0.06(0.02) ^c^	0.12(0.02) ^a^
2-Hexenal, (E)-	0.09(0.02) ^c^	0.09(0.02) ^c^	0.08(0.03) ^cd^	0.09(0.02) ^c^	0.20(0.02) ^a^	0.05(0.02) ^d^	0.14(0.02) ^b^	0.16(0.02) ^b^
Heptanal	n.d.	0.03(0.02) ^a^	0.05(0.02) ^a^	0.03(0.02) ^a^	0.04(0.02) ^a^	0.07(0.02) ^a^	0.06(0.02) ^a^	n.d.
Undecanal	0.04(0.03) ^a^	0.05(0.02) ^a^	0.04(0.02) ^a^	0.06(0.03) ^a^	0.08(0.02) ^a^	0.08(0.02) ^a^	0.08(0.02) ^a^	0.08(0.02) ^a^
Octanal	7.27(0.04) ^d^	7.18(0.03) ^d^	6.97(0.04) ^e^	6.52(0.05) ^f^	8.23(0.04) ^c^	8.96(0.06) ^b^	9.20(0.04) ^a^	9.23(0.03) ^a^
Nonanal	0.47(0.06) ^b^	0.34(0.03) ^cd^	0.36(0.02) ^cd^	0.43(0.04) ^bc^	0.63(0.03) ^a^	0.59(0.04) ^a^	0.59(0.02) ^a^	0.59(0.03) ^a^
Decanal	1.64(0.03) ^d^	1.36(0.05) ^e^	1.43(0.03) ^e^	1.62(0.03) ^d^	2.83(0.03) ^a^	2.28(0.05) ^c^	2.43(0.03) ^b^	2.30(0.04) ^c^
**Total**	9.58(0.20) ^d^	9.10(0.19) ^e^	8.99(0.18) ^f^	8.8(0.21) ^g^	12.13(0.18) ^c^	12.13(0.23) ^c^	12.56(0.17) ^a^	12.48(0.16) ^b^
**Terpenes and terpenoids**	**OJWP0**	**OJWP2.5**	**OJWP5**	**OJWP7.5**	**OPJ0**	**OPJ2.5**	**OPJ5**	**OPJ7.5**
Camphene	0.06(0.02) ^a^	n.d.	n.d.	n.d.	0.04(0.03) ^a^	0.08(0.02) ^a^	n.d.	n.d.
β-Pinene	0.49(0.02) ^c^	0.12(0.02) ^f^	0.25(0.03) ^d^	0.18(0.02) ^e^	0.62(0.03) ^b^	0.08(0.02) ^f^	0.62(0.04) ^b^	0.69(0.04) ^a^
Benzaldehyde	n.d.	n.d.	n.d.	n.d.	0.08(0.02) ^a^	0.07(0.03) ^a^	0.06(0.02) ^a^	0.07(0.02) ^a^
β-Myrcene	34.36(0.06) ^b^	34.88(0.18) ^a^	33.51(0.07) ^c^	32.49(0.08) ^d^	31.81(0.07) ^e^	31.17(0.10) ^f^	31.06(0.08) ^f^	31.00(0.12) ^f^
Limonene	34.18(0.04) ^c^	35.08(0.06) a	34.87(0.06) b	33.87(0.07) d	23.02(0.10) f	25.02(0.07) e	23.16(0.09) f	23.08(0.10) f
β-cis-Ocimene	0.86(0.02) ^c^	0.73(0.02) ^d^	0.71(0.02) ^d^	0.83(0.03) ^c^	1.05(0.04) ^b^	1.11(0.03) ^a^	1.06(0.03) ^ab^	1.03(0.03) ^b^
α-Phellandrene	n.d.	n.d.	0.06(0.02) ^ab^	0.03(0.01) ^b^	0.05(0.02) ^ab^	0.09(0.03) ^a^	0.07(0.03) ^ab^	n.d.
.gamma.-Terpinene	1.98(0.02) ^e^	1.91(0.02) ^e^	1.97(0.04) ^e^	2.20(0.04) ^d^	2.89(0.07) ^bc^	3.08(0.05) ^a^	2.81(0.06) ^c^	2.97(0.05) ^ab^
Terpinolene	0.11(0.02) ^b^	0.10(0.02) ^b^	0.11(0.02) ^b^	0.13(0.03) ^b^	0.21(0.02) ^a^	0.12(0.02) ^b^	n.d.	n.d.
α-Pinene	6.90(0.04) ^g^	6.37(0.03) ^h^	7.24(0.04) ^f^	7.82(0.06) ^e^	12.11(0.07) ^a^	10.27(0.06) ^d^	10.66(0.08) ^c^	11.36(0.08) ^b^
β-Linalool	0.64(0.02) ^e^	0.65(0.03) ^e^	0.61(0.02) ^e^	0.75(0.05) ^d^	0.53(0.05) ^f^	1.21(0.03) ^c^	1.53(0.03) ^b^	1.86(0.04) ^a^
(+)-4-Carene	1.24(0.02) ^b^	1.05(0.03) ^b^	1.07(0.04) ^b^	1.26(0.03) ^b^	1.69(0.06) ^a^	1.78(0.05) ^a^	1.73(0.04) ^a^	1.27(0.03) ^b^
Benzene, 2-ethenyl-1,3-dimethyl-	0.22(0.03) ^ab^	0.16(0.02) ^b^	0.22(0.03) ^ab^	0.22(0.03) ^ab^	0.27(0.03) ^a^	0.26(0.03) ^a^	0.23(0.03) ^a^	0.21(0.03) ^ab^
β-Terpineol	0.03(0.03) ^e^	0.14(0.03) ^d^	0.03(0.02) ^e^	0.05(0.03) ^e^	0.13(0.02) ^d^	0.23(0.02) ^c^	0.57(0.03) ^b^	0.79(0.03) ^a^
α-Terpineol	0.23(0.04) ^c^	0.19(0.03) ^cd^	0.15(0.03) ^de^	0.13(0.02) ^e^	0.33(0.02) ^b^	0.24(0.03) ^c^	0.40(0.02) ^b^	0.86(0.03) ^a^
α-Citral	n.d.	n.d.	n.d.	n.d.	0.19(0.02) ^a^	0.05(0.03) ^b^	n.d.	n.d.
Copaene	0.09(0.02) ^cd^	0.09(0.02) ^bcd^	0.06(0.03) ^d^	0.07(0.02) ^d^	0.19(0.03) ^a^	0.13(0.03) ^abc^	0.17(0.03) ^a^	0.15(0.04) ^ab^
1,3,8-*p*-Menthatriene	0.11(0.02) ^ab^	0.06(0.03) ^b^	0.13(0.02) ^a^	0.13(0.02) ^a^	0.15(0.03) ^a^	0.16(0.03) ^a^	0.14(0.03) ^a^	0.16(0.02) ^a^
Limonene epoxide	0.14(0.03) ^a^	0.15(0.03) ^a^	0.14(0.03) ^a^	0.14(0.03) ^a^	0.14(0.03) _a_	0.15(0.04) ^a^	0.20(0.03) ^a^	0.15(0.02) ^a^
Caryophyllene	0.13(0.03) ^b^	0.13(0.03) ^b^	0.08(0.02) ^c^	0.10(0.02) ^bc^	0.25(0.03) _a_	0.12(0.03) ^bc^	0.22(0.02) ^a^	0.20(0.02) ^a^
α-Caryophyllene	0.03(0.02) ^d^	0.08(0.02) ^cd^	0.08(0.02) ^cd^	0.08(0.02) ^cd^	0.12(0.03) ^abc^	0.13(0.03) ^ab^	0.17(0.03) ^a^	0.11(0.03) ^bc^
β-Elemene	n.d.	0.05(0.02) ^d^	0.07(0.03) ^bcd^	0.06(0.03) ^cd^	0.11(0.02) ^abc^	0.12(0.03) ^ab^	0.13(0.03) ^a^	0.10(0.03) ^abcd^
Naphthalene, 1.2,3,5,6,7,8,8a-octahydro-1.8a-dimethyl-7-(1-methylethenyl)-, [1R-(1.alpha.,7.beta.,8a.alpha.)]- //Valencene	1.33(0.03) ^f^	2.73(0.08) ^d^	2.42(0.05) ^e^	2.67(0.05) ^d^	4.12(0.08) ^b^	4.10(0.06) ^b^	4.29(0.07) ^a^	3.84(0.02) ^c^
2-Cyclohexen-1-one, 2-methyl-5-(1-methylethenyl)-, (R)-// (-)-Carvone	0.90(0.03) ^e^	1.00(0.03) ^d^	1.41(0.04) ^b^	1.80(0.05) ^a^	0.85(0.06) ^e^	0.29(0.04) ^f^	1.40(0.06) ^b^	1.21(0.02) ^c^
2-Cyclohexen-1-one, 3-methyl-6-(1-methylethenyl)-, (S)-	0.16(0.03) ^e^	0.36(0.03) ^c^	1.04(0.03) ^b^	1.18(0.04) ^a^	0.08(0.02) ^f^	0.25(0.03) ^d^	0.12(0.03) ^ef^	0.17(0.02) ^e^
Naphthalene, 1.2,3,5,6,8a-hexahydro-4,7-dimethyl-1-(1-methylethyl)-, (1S-cis)-//delta.-Cadinene	0.10(0.03) ^e^	0.08(0.02) ^e^	0.08(0.02) ^e^	0.20(0.03) ^d^	0.32(0.03) ^bc^	0.36(0.03) ^ab^	0.41(0.03) ^a^	0.29(0.01) ^c^
(-)-α-Panasinsen	n.d.	n.d.	n.d.	n.d.	0.21(0.03) ^a^	0.24(0.03) ^a^	0.23(0.04) ^a^	0.20(0.02) ^a^
2,6-Octadien-1-ol, 3,7-dimethyl-, acetate, (Z)-//Nerol acetate	0.05(0.03) ^b^	n.d.	n.d.	0.05(0.03) ^b^	0.06(0.03) ^ab^	0.07(0.03) ^ab^	0.08(0.02) ^ab^	0.11(0.01) ^a^
1-Cyclohexene-1-carboxaldehyde, 4-(1-methylethenyl)-	0.04(0.03) ^d^	0.20(0.03) ^c^	0.12(0.03) ^c^	0.25(0.03) ^bc^	0.21(0.03) ^bc^	0.04(0.02) ^d^	0.37(0.04) ^a^	0.27(0.02) ^b^
β-Citral	n.d.	n.d.	0.03(0.03) ^a^	0.05(0.03) ^a^	0.06(0.03) ^a^	0.05(0.02) ^a^	0.06(0.02) ^a^	0.04(0.02) ^a^
**Total**	84.38(0.68) ^b^	86.31(0.82) ^a^	86.45(0.88) ^a^	86.74(0.90) ^a^	81.89(1.16) ^c^	81.07(1.08) ^c^	81.95(1.05) ^c^	82.19(0.80) ^c^
**Ketones**	**OJWP0**	**OJWP2.5**	**OJWP5**	**OJWP7.5**	**OPJ0**	**OPJ2.5**	**OPJ5**	**OPJ7.5**
Acetophenone	n.d.	0.04(0.02) ^c^	0.04(0.02) ^c^	0.04(0.02) ^c^	0.05(0.02) ^c^	0.06(0.03) ^c^	0.18(0.02) ^b^	0.53(0.03) ^a^
Ethanone, 1-(4-methylphenyl)-	0.11(0.03) ^bc^	0.11(0.03) ^bc^	0.11(0.03) ^bc^	0.15(0.04) ^b^	0.06(0.03) ^c^	0.05(0.04) ^c^	0.18(0.04) ^b^	0.46(0.05) ^a^
**Total**	0.11(0.03) ^c^	0.15(0.06) ^c^	0.15(0.05) ^c^	0.19(0.07) ^c^	0.11(0.05) ^c^	0.11(0.07) ^c^	0.36(0.06) ^b^	0.99(0.08) ^a^
**Acids**	**OJWP0**	**OJWP2.5**	**OJWP5**	**OJWP7.5**	**OPJ0**	**OPJ2.5**	**OPJ5**	**OPJ7.5**
Butanoic acid, methyl ester	0.59(0.08) ^b^	0.04(0.01) ^d^	0.06(0.02) ^d^	0.06(0.02) ^d^	1.20(0.02) ^a^	0.10(0.02) ^cd^	0.07(0.02) ^d^	0.15(0.03) ^c^
Dodecanoic acid	0.17(0.04) ^b^	0.14(0.02) ^b^	0.14(0.03) ^b^	0.13(0.03) ^bc^	0.07(0.02) ^c^	0.25(0.03) ^a^	0.26(0.04) ^a^	0.24(0.02) ^ab^
Tetradecanoic acid	0.61(0.08) ^a^	0.14(0.04) ^d^	0.27(0.01) ^c^	0.24(0.04) ^c^	0.14(0.04) ^d^	0.18(0.02) ^cd^	0.38(0.04) ^b^	0.24(0.03) ^c^
Butanoic acid, ethyl ester	1.89(0.07) ^c^	1.35(0.15) ^d^	1.37(0.04) ^d^	1.41(0.03) ^d^	2.33(0.07) ^b^	3.48(0.05) ^a^	1.95(0.06) ^c^	2.19(0.11) ^b^
Acetic acid, octyl ester	1.40(0.08) ^a^	0.78(0.03) ^b^	0.74(0.06) ^b^	0.80(0.04) ^b^	0.15(0.03) ^c^	0.05(0.01) ^d^	0.07(0.03) ^cd^	0.04(0.01) ^d^
**Total**	4.65(0.36) ^a^	2.45 (0.25) ^d^	2.58(0.16) ^cd^	2.63(0.16) ^cd^	3.87(0.18) ^b^	4.06(0.13) ^b^	2.71(0.19) ^cd^	2.86(0.20) ^c^
n.i.	0.09	0.73	0.58	0.47	0.15	0.99	0.55	0.08

The same letter in superscript within the column indicates homogeneous groups established by ANOVA (*p* < 0.05). n.i., not identified; n.d., not detected; OJWP, orange juice without pulp; OJP, orange juice with pulp; 0–7.5 % of RMD addition.

**Table 4:** RV coefficients obtained from the MFA.

	Sensory Colour	Sensory Taste	Sensory Texture	Sensory Flavour	Instrumental Colour	Instrumental Taste	Instrumental Texture	Instrumental Aroma	MFA
**Sensory colour**	1.000	0.058	0.093	0.025	0.175	0.083	0.099	0.278	0.294
**Sensory taste**	0.058	1.000	0.733	0.394	0.700	0.645	0.685	0.459	0.808
**Sensory texture**	0.093	0.733	1.000	0.253	0.732	0.691	0.498	0.346	0.737
**Sensory flavour**	0.025	0.394	0.253	1.000	0.617	0.527	0.464	0.205	0.605
**Instrumental colour**	0.175	0.700	0.732	0.617	1.000	0.886	0.784	0.489	0.927
**Instrumental taste**	0.083	0.645	0.691	0.527	0.886	1.000	0.711	0.312	0.834
**Instrumental texture**	0.099	0.685	0.498	0.464	0.784	0.711	1.000	0.816	0.890
**Instrumental aroma**	0.278	0.459	0.346	0.205	0.489	0.312	0.816	1.000	0.690
**MFA**	0.294	0.808	0.737	0.605	0.927	0.834	0.890	0.690	1.000

## Data Availability

The data presented in this study is available on request from the corresponding author. The data are not publicly available due to industrial privacy reasons.

## Supplementary Materials

The following supporting information can be downloaded at https://www.mdpi.com/article/10.3390/foods12214025/s1, Figure S1: Superimposed MFA representation of the juices. Each sample is represented by eight points grouping sensory and instrumental variables studied in the samples. Each juice representation considers the eight variables simultaneously.
