# Supplementary material for: Sensory Evaluation, Physico-Chemical Properties, and Aromatic Profile of Pasteurised Orange Juice with Resistant Maltodextrin
Source: Foods. 2023 Nov 3;12(21):4025. doi: 10.3390/foods12214025 (PMC10648707; doi:10.3390/foods12214025)
Supplement: Supplementary file 1 [file foods-12-04025-s001.zip › foods-2655708-supplementary.pdf]

foods

MDPI

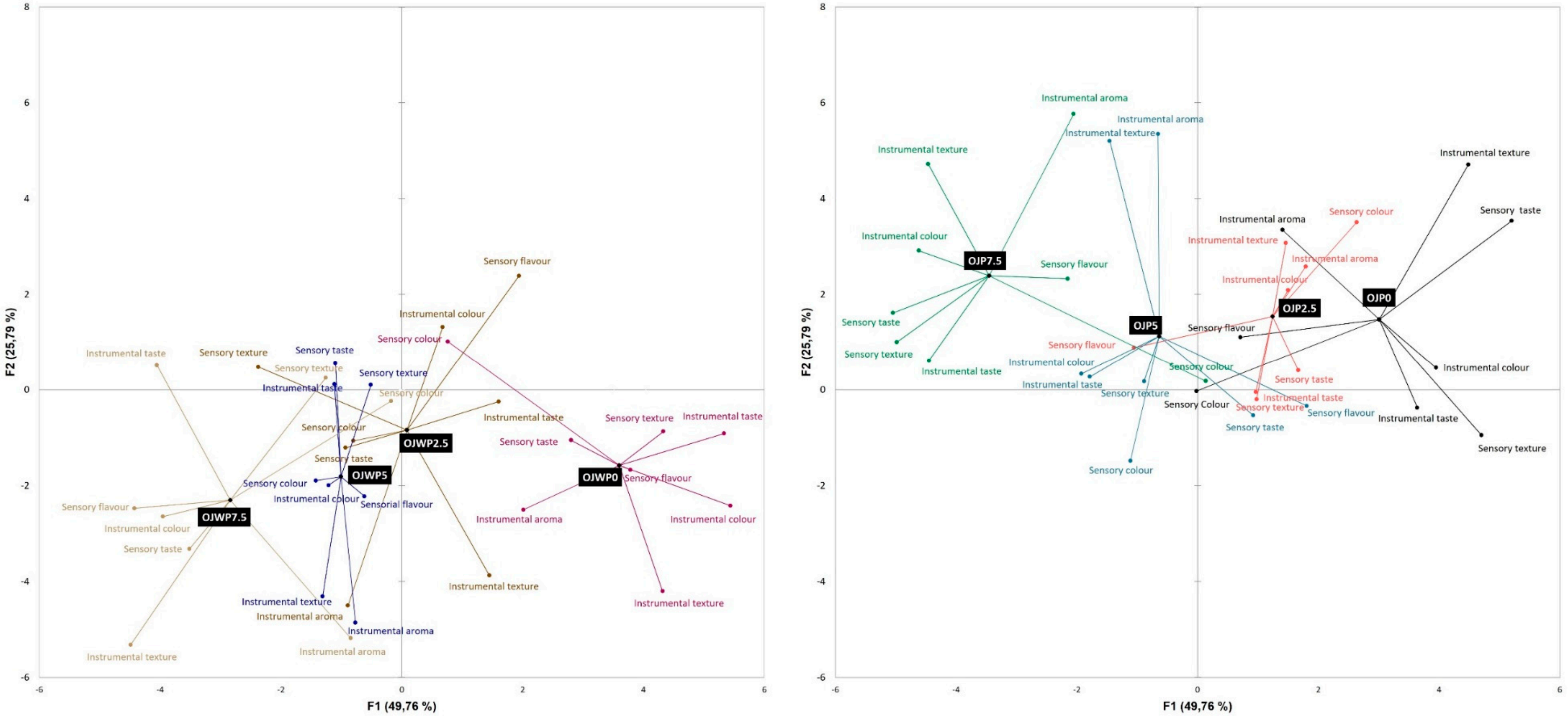

Figure S1: Superimposed MFA representation of the juices. Each sample is represented by eight points grouping sensory and instrumental variables studied in the samples. Each juice representation considers the eight variables simultaneously.
